# Telemedicine in Low-Resource Settings

**DOI:** 10.3389/fpubh.2015.00003

**Published:** 2015-01-21

**Authors:** Richard Wootton, Laurent Bonnardot

**Affiliations:** ^1^Norwegian Centre for Integrated Care and Telemedicine, University Hospital of North Norway, Tromsø, Norway; ^2^Faculty of Health Sciences, University of Tromsø, Tromsø, Norway; ^3^Fondation Médecins Sans Frontières, Paris, France; ^4^Department of Medical Ethics and Legal Medicine (EA 4569), Paris Descartes University, Paris, France

**Keywords:** telemedicine, low-resource settings, telehealth, health care, editorial

Telemedicine is a fuzzy term with several synonyms (telehealth, e-health, etc), which cover a wide range of topics, all concerning the delivery of health care at a distance. “Health care” itself is a broad concept, encompassing diagnosis and treatment of patients, education of staff, patients, and the general public, and administrative activities, such as collecting public health data, as well as research. All of these may be assisted by judicious use of telemedicine.

The main advantage of telemedicine is that it can improve access to health care, often by increasing the speed with which a specialist opinion can be obtained (e.g., tele-stroke) or by reducing the need to travel (e.g., teledermatology); in certain disciplines, evidence has also been obtained that telemedicine is cost-effective (1). Much of the experience with telemedicine in the last 20 years has concerned its application in high-income countries. In contrast, there has been relatively little use of telemedicine in low-income countries, which is surprising in view of the difficulties of accessing health care there. In those countries where telemedicine has been trialed, it seems to have worked well and a small number of programs have provided services for periods of 10 years or more (2). These long-running telemedicine programs have mainly used store-and-forward methods, although there has been some limited use of real-time video.

The present Research Topic focuses on *Telemedicine in Low-Resource Settings*, environments where it is always a challenge to provide patients with the best level of health care. The term “low-resource settings” covers most low-income countries, and also includes regions in middle- or high-income countries where under-served populations have difficulties in accessing specialists. The Research Topic documents real, practical experience with the use of telemedicine in low-resource settings and identifies research problems of current interest. This collection of articles shows the rich diversity of applications for telemedicine. Examples come from all over the world and from a range of clinical settings and medical specialties.

Mobile phones have great potential in the delivery of health care in low-resource settings. Patterson (3) developed a mobile-phone app to enable non-doctors to diagnose episodes as epileptic. In a pilot trial with health workers in Nepal who used the app in small numbers of patients, there were no false diagnoses. This represents a potential method of empowering health workers to help the millions of people in the resource-poor world with untreated epilepsy. Ndlovu et al. (4) conducted trials with mobile-phone telemedicine in Botswana, in four medical specialties: radiology, oral medicine, dermatology, and cervical cancer screening. The benefits reported by pilot project users were sufficient to convince the government to scale up the program, which is now in progress. Both senior management support and local “ownership” of the program are thought to be important for future success. Piette et al. (5) also reported on the importance of collaborating with the local ministry of health when scaling up a mobile telemedicine application in Bolivia. All these experiences reinforce the need to develop telemedicine by scaling it up from pilot projects, to do so in collaboration with local healthcare workers (rather than trying to impose telemedicine from above) and to enlist the support of the appropriate ministry of health.

One of the longer-running examples of telemedicine used in low-resource settings is the RAFT network, which provides both educational and clinical services to centers in Africa and South America (6). The educational activities include the weekly delivery of video-lectures for continuing and postgraduate medical education. Much of this early video delivery depended on the use of satellite links, which are relatively expensive, and in recent years the RAFT program has begun to make use of low-bandwidth Internet connections. In South Africa, a tele-education network evolved from a failed government telemedicine program (7). Over 1000 h of videoconferenced lectures are delivered each year in KwaZulu-Natal, using ISDN transmission. Finally, the EHAS group has provided video-based telemedicine services in South America (8). In order to secure sufficient bandwidth for the delivery of video, they have developed long-range WiFi transmission.

An alternative method of transmitting video for telemedicine is to make use of free or low-cost web-based tools. For example, Jefee-Bahloul (9) conducted a pilot trial of telepsychiatry in Jordan using Skype, while Adambounou et al. (10) used the file transfer facilities of the LogMeIn web service for tele-ultrasound between Togo and France.

It is clear from these reports that video telemedicine is possible in low-resource environments, but it is also the case that non-real-time (store-and-forward) telemedicine is more common in these settings, not only because it is usually cheaper but also because the non-synchronous nature of the interaction between the parties makes it easier to organize. The longest-running such network is probably operated by the US military in the Pacific, which has used email and web-based communication in the Pacific Island Health Care Project since the late 1990s. As Person reports (11), teleconsultation has enabled local treatment in the Pacific islands, without necessarily requiring transfer to the major medical center on Hawaii; many of the cases were pediatric. Andronikou (12) reviewed his experience of pediatric teleradiology with three different store-and-forward programs. He concluded that teleradiology offers the potential to alleviate radiologist shortages in under-served areas, but that there are many challenges to designing an adequate process.

Médecins Sans Frontières (MSF), an organization that works mainly in low-resource settings, developed its own telemedicine tool based on the Collegium Telemedicus model (13). The aim was a system that would improve the primary-specialty care interface and allow their field doctors to obtain an expert opinion within a few hours, wherever they were located in the world. Based on a retrospective analysis and user survey, Bonnardot et al. (14) provide a general overview of the system and the user perceptions of it. The three main specialties used in the network are radiology, pediatrics, and dermatology, which were reviewed by Halton et al. (15), Delaigue et al. (16), and Martinez Garcia et al. (17), respectively.

The MSF experience, and that of others reported here, suggests that store-and-forward networks are clinically useful, sustainable, and potentially cost-effective. It is also clear that there is still lingering skepticism from some healthcare staff about the adoption of telemedicine into routine practice. Apparently, telemedicine is sometimes viewed as a threat or a competitor to conventional ways of working. Yet, telemedicine is simply another tool for assisting in the delivery of health care, and in low-resource settings there is often no other way to access the required resources.

As telemedicine matures to become a routine service in low-resource settings, it will become increasingly important to evaluate the quality of service being delivered and to demonstrate that this is being maintained. There is almost no published work about quality assurance in this context, and the present Topic contains three papers, which explore different aspects of this new area (18–20). While providing initial demonstrations of feasibility, each raises a number of questions for future research.

In summary, this e-book provides vignettes illustrating (largely successful) telemedicine projects of widely different kinds in various low-resource settings. It is worth noting that solutions that are found to overcome the huge constraints imposed by low-resource settings may also be useful in middle- or high-income countries. The common themes are that success depends on expanding from small pilot projects using a “bottom-up” approach with engagement of local health workers, yet also requires senior management and government support. The research agenda for the future requires us to document the cost-effectiveness of these programs, and as telemedicine matures, to demonstrate that quality improvement activities can be incorporated in the same way as is done in many other areas of health care. We can expect that in the future, the use of telemedicine – practising health care at a distance – will become a norm. Indeed, we expect that it will become so common as to be unremarkable, that the prefix *tele-* will disappear, and that all telemedicine work will be considered as part of usual practice.

## Conflict of Interest Statement

The authors declare that the research was conducted in the absence of any commercial or financial relationships that could be construed as a potential conflict of interest.
